# Prevalence and correlates of reproductive coercion in the months before pregnancy: cross-sectional findings from pregnant women in Ethiopia

**DOI:** 10.1186/s12978-025-02192-x

**Published:** 2025-11-12

**Authors:** Jessica L. Dozier, Linnea A. Zimmerman, Robel Yirgu, Nancy Perrin, Solomon Shiferaw, Shannon N. Wood

**Affiliations:** 1 Department of Population, Family and Reproductive Health, Johns Hopkins Bloomberg School of Public Health, Baltimore, MD 21205 USA; 2 School of Public Health, Addis Ababa University, Addis Ababa, Ethiopia; 3 School of Nursing, Johns Hopkins University, Baltimore, MD 21205 USA; 4 University of Michigan School of Nursing, 400 N. Ingalls St., MI 48109 Ann Arbor, USA

**Keywords:** Reproductive coercion, Pregnancy coercion, Sub-Saharan Africa, Correlates, Intimate partner violence

## Abstract

**Background:**

Reproductive coercion (RC), a form of intimate partner violence involving pressure, threats, or manipulation to control women’s reproductive decisions—including contraceptive use and pregnancy—poses significant threats to women’s health and rights by constraining their ability to make autonomous choices. This study examined the prevalence and correlates of past-year RC among pregnant Ethiopian women.

**Methods:**

Cross-sectional data collected between October and December 2019 from the Performance Monitoring for Action-Ethiopia population-based cohort of pregnant women (*N* = 2169) were used. Past-year RC was assessed as a binary (any RC) and categorical (none, less severe, more severe) variable. Descriptive analyses were conducted to examine RC and sample characteristics. Estimated marginal effects were used to predict the probability of RC in the year before pregnancy. Binary and multinomial logistic regression were used to identify correlates of RC.

**Results:**

27.3% of pregnant women reported any past-year RC (16.2% less severe; 11.1% more severe). The adjusted probability of any RC in the year before pregnancy was 32.6% (95% CI: 26.1–39.2; 18.2% less severe; 14.3% more severe). Risk factors for any RC included higher household wealth (aOR _highest quintile_ = 2.57, 95% CI: 1.50–4.13) and residence in Amhara (aOR = 1.82, 95% CI: 1.21–2.75); protective factors included having 2 + children (aOR_2-3 children_ = 0.57, 95% CI: 0.41–0.80; aOR_4+ children_ = 0.49, 95% CI: 0.34–0.72), or more than secondary education (aOR = 0.39, 95% CI: 0.22–0.64). Higher household wealth relative to the poorest households was a risk factor for less severe RC (aRRR _highest quintile_ = 4.16, 95% CI: 2.32–7.44); whereas residing in Afar (aRRR = 0.35, 95% CI: 0.13–0.94) and having 2 + children (aRRR_2-3 children_ = 0.60, 95% CI: 0.42–0.87; aRRR_4+ children_ = 0.42, 95% CI: 0.27–0.68) were protective. Living in Amhara (aRRR = 2.40, 95% CI: 1.34–4.04), cohabitating (aRRR = 2.04, 95% CI: 1.06–3.94), and having a previous marriage or cohabitating relationship (aRRR = 1.84, 95% CI: 1.22–2.78) were associated with increased risk of more severe RC, whereas having 2 + children (aRRR_2-3children_ = 0.54, 95% CI: 0.32–0.91) or more than secondary education (aRRR = 0.16, 95% CI: 0.07–0.40) were protective.

**Conclusions:**

In Ethiopia, many women experience RC before pregnancy. Having two or more children and more than a secondary education are protective against RC. Risk factors for the most severe forms of RC include residing in Amhara, greater household wealth, cohabitation, and previous marriage or cohabitating relationships. Findings highlight the need for targeted interventions that address modifiable factors in high-risk populations and settings, such as engaging boys and men to prevent RC, and integrating RC response into pregnancy care to enhance women’s reproductive autonomy.

## Abstract (plain language summary)

Reproductive coercion (RC) is a form of intimate partner violence where a husband or partner uses pressure, threats, or manipulation to control a woman’s reproductive choices, such as whether or when to use contraception or become pregnant. RC is a serious public health issue that affects women’s health and limits their ability to make independent decisions about contraception and pregnancy. We examined how common RC is among pregnant women in Ethiopia and what factors are associated with it. A total of 2,169 pregnant women from six regions were surveyed about whether, in the past year, their husband or partner engaged in any of the following pregnancy promoting behaviors: 1) told her not to use family planning; 2) said he would leave her if she didn’t get pregnant; 3) said he would have a baby with someone else if she didn’t get pregnant; 4) took away her family planning or kept her from going to the clinic; or 5) hurt her physically because she did not get pregnant. We measured RC in two ways: with a binary variable to indicate whether RC happened at all in the past year and a categorical variable to show the severity of RC (none, less severe, more severe). An estimated one-third of women experienced RC in the year before becoming pregnant, with 14.3% facing the most severe forms. The likelihood of experiencing the most severe forms of RC was higher among women living in Amhara, those who were cohabiting, and those who had a previous marriage or cohabitating relationship before their current partner. On the other hand, having more education and more children were protective against more severe RC. Results suggest many women in Ethiopia experience RC before pregnancy and highlight the need for interventions that address modifiable risk factors, educate boys and men, and integrate RC response strategies into pregnancy care to protect women's reproductive autonomy.

## Introduction

Intimate partner violence (IPV) is a violation of fundamental human rights [1–3] and a significant contributor to poor sexual and reproductive (SRH) outcomes among women and girls globally. [4–9] Reproductive coercion (RC), a prevalent form of IPV, occurs when a partner uses pressure, threats, physical or sexual violence, or manipulation to interfere with women’s reproductive autonomy. [10] RC poses a direct threat to women’s health and their right to self-determination. [11–13] In sub-Saharan Africa, population-based studies estimates past-year RC among women of reproductive age ranges from 3.1% in Niger to 20.3% in Kongo Central, Democratic Republic of Congo. [14] In high fertility settings, pronatalist preferences of partners and relatives, as well as social expectations of women to prove fertility shortly after marriage, may drive male partners to perpetrate RC. [15–18] Studies in sub-Saharan Africa and Asia have linked RC to decreased contraceptive use [19, 20], greater risk of unintended pregnancy [20], and shorter interpregnancy intervals [21], indicating that women who experience partner interference in their reproductive health have diminished control over their fertility. [12] Emerging evidence also suggests that pre-pregnancy RC is associated with adverse maternal and child health outcomes, including increased risk of physical/sexual IPV during pregnancy [22], prenatal maternal distress [23], having a low birth weight neonate [24], and a reduced likelihood of infant immunization. [22]

Pregnant women are a key population for understanding RC and its influence on women’s health and well-being. However, no research has yet examined correlates of pre-pregnancy RC, even though pregnant women may suffer the most deleterious consequences from recent RC—coercion into an unwanted pregnancy. Associations between RC and unintended pregnancy [20, 25] suggest that RC limits women’s ability to make autonomous decisions about *whether and when* to become pregnant. [12, 20, 26] Moreover, RC is associated with ongoing IPV during pregnancy [22] and adverse maternal and child health outcomes [22–24]. Identifying correlates of RC before pregnancy enables targeted interventions to prevent its occurrence, thereby reducing the likelihood of unintended pregnancy. Additionally, responding to partner-perpetrated RC in pronatalist settings may help mitigate closely spaced pregnancies [27] and enable women to freely decide about postpartum contraceptive use and pregnancy spacing.

Sociodemographic risk factors for RC in general population samples in sub-Saharan Africa include lower parity [14, 19], lower education levels [14], poverty [14], cohabitation without marriage [14], and having a partner with multiple wives [14, 28]. In Ethiopia, Wood and colleagues found that nulliparous women had a greater risk of experiencing RC than women with at least one child [19]. Additionally, women who never attended school were at higher risk of RC than those with a secondary or higher level of education [19]. When RC was further examined by severity—with partner discouragement of family planning considered “less severe RC” and behaviors involving specified coercive intent to force women into becoming pregnant considered “more severe RC”—residence in an urban area was associated with an elevated risk of less severe RC but was protective against the most severe RC [19]. Likewise, cohabitating relationships, as opposed to marriage, were associated with a decreased risk of less severe RC but an increased risk of more severe RC [19]. These findings indicate that drivers of RC may vary by behavior severity in Ethiopia. Therefore, delineation of partner verbal discouragement of family planning from explicit interference or sabotage can help uncover specific intentions tied to different forms of RC, which range in severity and consequence. Explicit interference or sabotage directly undermines a woman’s ability to act on their reproductive choices and suggests a more intentional and immediate effort to override her reproductive choices.

Understanding correlates of RC, particularly in settings characterized by high fertility, pronatalist and gender inequitable norms, low contraceptive use, and high rates of unintended pregnancy, is essential for the development of evidence-based strategies for RC prevention and response. Furthermore, examining correlates of pre-pregnancy RC holds particular significance in the Ethiopian context where social norms incite significant pressure on women to bear children shortly after marriage and to have many children [11]. Gender and power disparities further enable male partners to assert control over decisions concerning contraception, the timing and spacing of pregnancies, as well as the overall family size of the couple [29, 30]. Understanding the correlates of RC most proximal to pregnancy is key for efforts to support women’s autonomy over their reproductive choices and prevent RC and unintended pregnancies resulting from RC. Further research is needed to identify specific correlates of partner-perpetrated RC before pregnancy to inform prevention efforts and support services for women at the highest risk of unintended pregnancy. The present study aims to examine the prevalence and sociodemographic correlates of partner-perpetrated RC in the past year among a population-based sample of pregnant Ethiopian women.

## Methods

### Study setting

In Ethiopia, the second most populous country in sub-Saharan Africa [31], discordant fertility intentions within couples are common, often characterized by male partners expressing pronatalist preferences [28, 32]. Ethiopian men typically have a larger ideal family size than women; married men and women aged 15–49 report wanting an average of 5.5 children and 4.9 children, respectively [31]. Regardless of the number of living children, women are more likely than men to want to stop having children; 36% of currently married women aged 15–49 want to limit childbearing compared to 26% of men [31]. On average, Ethiopian women have one more child than they want, with a wanted fertility rate of 3.6 children versus a total fertility rate of 4.6 children. [31] Moreover, two in five (42%) pregnancies each year are unintended [33].

Traditional norms often emphasize men’s authority and dominance in decision-making regarding sex and reproductive health [30, 34, 35], which can create an environment where male partners exert pressure or control over contraceptive use and pregnancy-related decisions [11, 16, 36]. Approximately one in five women of reproductive age have experienced RC in the past year [19]. Gender and power imbalances are further evidenced by the fewer than half of Ethiopian women who report they could refuse sex and less than one-third who report they could ask their husbands to use a condom [31]. High rates of IPV [31, 37] and child marriage [31] further reflect significant gender power imbalances present in this context. Over one-third (37%) of married women aged 15–49 use modern contraceptive methods, and 20% have an unmet need for family planning [38].

### Data source and study design

Performance Monitoring for Action Ethiopia (PMA-Ethiopia) is a collaborative research initiative between Addis Ababa University, the Johns Hopkins Bloomberg School of Public Health, and the Ethiopian Federal Ministry of Health (2018–2023) [39]. PMA-Ethiopia aims to generate timely data on reproductive, maternal, and newborn health indicators using a combination of regionally and nationally representative cross-sectional and longitudinal surveys. The cross-sectional data used for the present study were collected between October and December 2019 as part of baseline surveys for PMA-Ethiopia 2019–2021, a population-based cohort of pregnant and recently postpartum women followed through the first-year postpartum. The goal of the cohort was to evaluate coverage and comprehensiveness of the reproductive, maternal, and newborn health continuum and identify correlates of care-seeking.

PMA-Ethiopia employed multistage stratified cluster sampling, wherein households were selected from 217 clusters or enumeration areas (EAs) across six regions that collectively represent 91% of Ethiopia’s population: Addis Ababa, Afar, Amhara, Oromia, Tigray, and the Southern Nations Nationalities, and Peoples Region (SSNP) [39]. EAs were stratified by region, with additional urban/rural residence stratification in Amhara, Oromia, SNNP, and Tigray.^1^ No urban/rural stratification was applied to Addis Ababa or Afar because these regions are almost exclusively urban and rural, respectively. All EAs were selected with probability proportional to size. A census of all households in selected EAs identified women ages 15–49 who were regular household members. All women who self-reported being currently pregnant or less than six weeks postpartum were eligible for cohort enrollment [39]. A total of 2,855 women (2,239 currently pregnant and 616 less than six weeks postpartum) consented to participate, yielding a response rate of 99.6%. Local interviewers, trained in ethical best practices for conducting research on violence against women [40], administered surveys using Open Data Kit Collect, an open-source data collection software used on mobile phones [39].

### Participants

The initial study sample for the PMA-Ethiopia cohort baseline interviews consisted of n = 2,855 pregnant or recently postpartum women at enrollment. Analyses were restricted to women who were pregnant at baseline, excluding the 616 women who were postpartum at enrollment because the independent variable of interest was pregnancy-promoting RC occurring up to 12 months before the current pregnancy. Including postpartum women could introduce ambiguity, as past-year RC might have occurred during the postpartum period, complicating interpretation of correlates of pre-pregnancy RC. In addition, restricting the sample to pregnant women ensures the RC recall period fully reflects the pre-pregnancy timeline. Additionally, women who were not married or living with a partner as if married were excluded (n = 57), as RC items were asked exclusively of partnered women. After examining patterns of missingness for RC items, a complete-case approach was employed, resulting in the exclusion of 13 observations with missing RC data. The final analytic sample included 2,169 pregnant women ages 15–49, married or cohabitating, with complete RC data.

### Ethics

Institutional Review Boards at Addis Ababa University (Ref: AAUMF 01–008) and the Johns Hopkins Bloomberg School of Public Health (FWA00000287) approved study procedures. Following the National Research Ethics Review guidelines in Ethiopia, all participants provided verbal informed consent. Parental consent was waived for participants ages 15–17, in accordance with the Ethiopian National Ethics Guidelines, given data collection covered sensitive topics, including SRH [41]. PMA-Ethiopia adhered to best practices for research on violence against women [40], including training interviewers to ensure respondent privacy and monitor for emotional distress. Support and referral protocols were in place for all women, regardless of violence disclosure, including the administration of a universal upset screener and referrals to health centers for violence and reproductive health support.

### Measures

Past-year RC was measured using five questions adapted from the pregnancy coercion sub-scale of the Reproductive Coercion Scale [25, 42]. Items from the condom manipulation sub-scale were omitted from PMA-Ethiopia due to the low prevalence of condom use (< 1%) among married women of reproductive age [43]. Pregnant women were asked whether, in the past 12 months, their husband/partner engaged in any of the following pregnancy-promoting behaviors: (1) told her not to use family planning, (2) said he would leave her if she didn’t get pregnant, (3) told her he would have a baby with someone else if she didn’t get pregnant, (4) took away her family planning or kept her from going to the clinic, or (5) hurt her physically because she did not get pregnant. A priori confirmatory factor analysis indicated that these items measure one latent pregnancy coercion factor (eigenvalue = 1.60) with moderate internal consistency (Cronbach’s alpha = 0.64). Except for the item “hurt you physically because you did not agree to get pregnant,” (factor loading = 0.31), all items had factor loadings greater than 0.4. The “hurt” item was retained due to its theoretical importance in capturing the most severe form of RC—physical harm resulting from disagreement about getting pregnant—which was not captured by the other items.

RC was assessed as both a binary variable (any affirmative response indicating any past-year RC) and a categorical variable with three levels: no RC, less severe RC, and more severe RC. The categorical variable was designed to capture differences in levels of RC severity, distinguishing verbal discouragement of contraception (item 1) from explicit interference and sabotage (items 2–5), consistent with prior research in Ethiopia [19, 44]. Specifically, an affirmative response to item 1 only (i.e., told you not to use family planning) was classified as less severe RC, as this may occur without explicit coercive intent. Affirmative responses to items 2–5, which involve active interference, threats, or control over contraceptive use and pregnancy, were classified as more severe RC. This classification reflects differences in coercive intent and potential consequences, while maintaining alignment with previous studies [9, 43].

All independent variables were assessed during pregnancy. Correlates were considered based on theory and existing literature [14, 19, 45, 46], including, region (Addis Ababa, Amhara, Afar, Oromia, SNNP, or Tigray); residence (urban/rural); household wealth quintile (categorized into lowest, lower, middle, higher, highest quintiles); relationship status (married or living with a man as if married); relationship history (whether a woman was previously married/lived with a partner or if the current relationship is her first such union); polygynous union (yes/no); age (grouped into 15–19, 20–24, 25–29, 30–34, 35–39, 40–49); parity (categorized as nulliparous, 1 child, 2–3 children, or 4 or more children); religion (Orthodox, Muslim, or Protestant/Other); and highest level of formal schooling attended (never attended, primary, secondary, or more than secondary). The wealth quintile variable was derived from household assets and previously calculated at the country level by the PMA-Ethiopia survey team using principal components analysis [39].

Since past-year RC risk decreases with advancing gestational age, as women are no longer at risk of pregnancy coercion once they become pregnant, self-reported gestational age (ranging from 2 to 9 months) was included as a covariate in multivariable models to account for differential exposure time to RC.

### Statistical analyses

Descriptive statistics examined the distribution of sociodemographic characteristics and prevalence of RC. Missing values for gestational age (n = 3) were imputed using median imputation (median = 5). Design-based F-statistics tested for significant differences between women who did and did not experience RC. A multivariable logistic regression model was run to examine correlates of any RC (binary outcome; adjusted model 1). A multivariable multinomial regression model was run to examine correlates of each level of RC severity (categorical outcome; adjusted model 2). We estimated associations using binary logistic regression for any RC, reporting odds ratios (ORs), and multinomial logistic regression for RC severity, reporting relative risk ratios (RRRs), as these are the standard effect measures for each model type. Variable selection for adjusted models was guided by theoretical relevance and bivariate associations with a significance threshold of p < 0.2. Age and residence were ultimately excluded from adjusted models due to collinearity with parity (0.71) and wealth (−0.74), respectively.

After conducting exploratory analyses to confirm marginal effects decreased with gestational age, post-estimation analysis using the Stata “margins” command was conducted to obtain the predicted probability of experiencing past-year RC, assuming all women were at risk of RC for the entire 12 months (i.e., when not pregnant or gestational age = 0). This allowed us to describe the probability of RC if all women in the sample had 12 months of exposure time for RC, while other independent variables were held at their means.

All analyses were performed using Stata 16.1, employing the “*svy*” command prefix to account for clustering of EAs and unequal probability of selection. Analyses were weighted (using weights constructed based on the selection probabilities of EAs and non-response within EAs) to represent all pregnant women ages 15–49 in the six study regions.

## Results

### Sample characteristics

The study included a total of 2,169 pregnant women with an average gestational age of 5.2 months (Table 1). Over one-half of women (52.7%) were between the ages of 20–29. The majority of women resided in Amhara (20.2%), SNNP (24.0%), or Oromia (43.4%), and most lived in rural areas (78.6%). Nearly all women in the sample were married (97.1%), and 12.8% had previously been in a marital or cohabitating relationship before their current partner. Additionally, one in ten (9.3%) women reported their partner had other wives. A quarter (23.4%) of women had not previously given birth.

**Table 1:** Sociodemographic characteristics of the analytic sample, PMA-Ethiopia 2019 (*N*=2169)

	**Weighted %**	**Weighted N**	**Unweighted N**
Region			
Addis Ababa	3.6	78	200
Afar	2.0	44	190
Amhara	20.2	438	361
Oromia	43.4	941	534
SNNP	24.0	521	528
Tigray	6.8	147	356
Residence			
Urban	21.4	463	812
Rural	78.6	1706	1357
Household wealth			
Lowest quintile	19.8	430	389
Lower quintile	19.7	426	326
Middle quintile	21.1	458	350
Higher quintile	19.8	429	403
Highest quintile	19.6	426	701
Relationship status			
Married	97.1	2106	2088
Living with partner as if married	2.9	63	81
Relationship history			
Current relationship is first union	87.2	1891	1902
Woman previously married/cohabitated	12.8	278	267
Polygyny			
No	90.7	1967	1976
Yes	9.3	202	193
Age			
15–19	10.6	231	204
20–24	23.5	509	533
25–29	29.2	634	677
30–34	19.3	418	411
35–39	13.2	286	269
40–49	4.2	91	75
Parity			
Nulliparous	23.4	508	568
1 child	19.4	421	450
2–3 children	26.2	569	573
4 or more children	30.9	671	578
Religion			
Orthodox	37.7	818	961
Muslim	33.9	735	658
Protestant/Other^a^	28.4	616	550
Education			
Never attended	42.1	912	838
Primary	39.7	862	780
Secondary	11.0	238	318
More than secondary	7.2	157	233
Gestational age (months), mean (SD)	5.2 (2.19)	-	5.31 (2.18)
Total	100.0	2169	2169

### Prevalence and predicted probability of past-year reproductive coercion among pregnant women

**Fig. 1:**
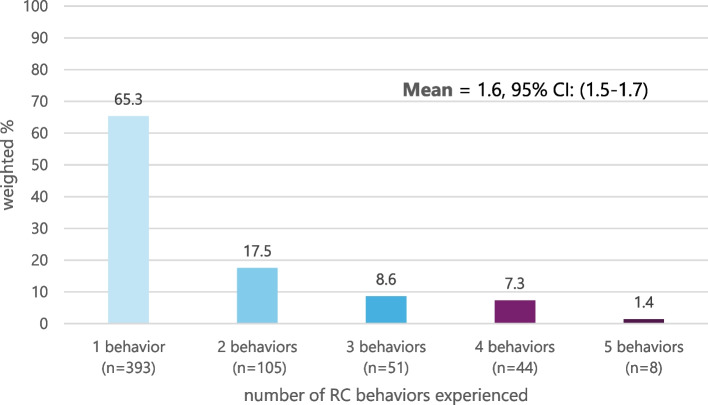
Number of pregnancy-promoting RC behaviors reported in the past 12 months by pregnant women, *n* = 602. *Note*. Of women who reported 1 RC behavior, 10% (n = 39/393) experienced a behavior classified as “more severe RC.” 84.6% of more severe RC included multiple behaviors (not shown; mean = 2.4)

Approximately three in ten pregnant women (27.3%) reported experiencing any RC in the past year (Table 2). The most prevalent form of RC was a partner telling the woman not to use family planning (24.4%), followed by threats to leave the relationship if she did not become pregnant (7.6%). Figure 1 illustrates the number of RC behaviors reported by women. On average, women who experienced RC reported 1.6 different behaviors (95% CI: 1.5–1.7), with the number of behaviors experienced ranging from 1 to 5.

**Table 2:** Prevalence and predicted probability of experiencing RC in the 12 months before pregnancy, N = 2169

	N	% (95% CI)	Predicted Probability of RC in 12 months before pregnancy	
			Unadjusted Pr (95% CI)	Adjusted^a^ Pr (95% CI)
Individual Items *In the past 12 months, has your husband/partner:*				
1. Told you not to use family planning	536	24.4 (21.6–27.5)	31.4 (23.5–39.2)	30.1 (23.2–36.9)
2. Said he would leave you if you didn’t get pregnant	159	7.6 (6.0–9.5)	10.6 (6.9–14.2)	10.1 (6.8–13.4)
3. Told you he would have a baby with someone else if you didn’t pregnant	102	5.0 (3.9–6.4)	6.3 (3.2–9.4)	6.1 (3.1–9.2)
4. Took away your family planning or kept you from going to the clinic	113	6.2 (4.7–8.0)	9.4 (4.6–14.2)	9.1 (4.8–13.4)
5. Hurt you physically because you did not agree to get pregnant	24	1.5 (0.9–2.2)	1.7 (0.2–2.2)	2.1 (0.07–4.2)
Composite Indicators				
Any experience of RC (items 1–5)	602	27.3 (24.6–30.8)	33.9 (26.7–41.1)	32.6 (26.1–39.2)
RC severity				
No RC	1571	72.7 (69.5–75.7)	66.6 (59.3–73.9)	67.4 (60.9–74.0)
Less severe RC (item 1 only)	365	16.2 (14.0–18.7)	19.1 (13.2–25.1)	18.2 (13.0–23.5)
More severe RC (items 2–4)	233	11.1 (9.1–13.5)	14.7 (9.7–19.7)	14.3 (9.7–18.9)

Predicted probability of RC at gestational age = 0, which accounts for women’s varying time at risk of RC by gestational age given the past-year timeframe of RC

*CI* Confidence interval

^a^Adjusted for region, wealth, relationship status, relationship history, parity, religion, education

When analyzing RC by categorical severity, 16.2% of women experienced only discouragement of family planning, classified as less severe RC (Table 2), and 11.1% of women reported at least one behavior classified as more severe RC. Among those who faced more severe RC, the majority (84.6%) experienced multiple coercive behaviors in the past year, with an average of 2.4 different behaviors reported (data not shown).

Using women’s reports of RC shown above and holding independent variables at their means, we estimated the predicted probability of experiencing pregnancy-promoting RC in the 12 months preceding pregnancy (i.e., probability of experiencing RC if women had not been pregnant during the past 12 months and therefore, were at risk of RC for the full 12 months). The adjusted predicted probability of experiencing any RC in the 12 months before becoming pregnant was 32.6% (95% CI 26.1–39.2). Specifically, there was an 18.2% (95% CI 13.0–23.5) adjusted predicted probability of experiencing less severe RC and a 14.3% (95% CI 9.7–18.9) adjusted predicted probability of experiencing more severe RC in the 12 months leading up to pregnancy (Table 2).

In bivariate analyses (Table 3), experience of any RC in the past year significantly differed by region, residence, household wealth, relationship history, age, and parity. The prevalence of any past-year RC varied across regions, ranging from 15.6% in Afar to 35.9% in Amhara (*p* = 0.043). A marginally higher proportion of women residing in urban areas (32.4%) experienced any RC compared to those in rural areas (26.2%; *p* = 0.049). The experience of any RC also varied by household wealth, ranging from 18.1% among women living in the poorest households to 32.5% among women living in the wealthiest households; any RC increased as household wealth increased (*p* = 0.006). Approximately one-third (33.8%) of women with a history of previous marriages or cohabitating relationships reported experiencing any RC compared to a quarter (26.6%) of women in their first union (*p* = 0.019). Experience of any RC varied across the life course, with the highest prevalence reported among the youngest (34.5%) and oldest women (31.7%; *p* = 0.008). In addition, any RC prevalence decreased as parity increased, from 35.1% among nulliparous women to 21.5% among women with four or more children (*p* < 0.001). No significant differences in any RC were observed by relationship status, polygyny, religion, or education.

**Table 3:** Bivariate associations between sociodemographic characteristics and reproductive coercion (RC), by Experience of any RC and RC severity, (*N* = 2169)

	*Total N*	**ANY RC** (*binary RC*)				**RC SEVERITY** (*categorical RC*)					
		**No** *N* = *1567*	**Yes** *N* = *602*		**OR(95% CI)**	**None** *N* = *1567*	**Less Severe** *N* = *365*	**More Severe** *N* = *237*		**Less Severe** **RRR** **(95% CI)**	**More Severe** **RRR (95% CI)**
		*Weighted %*		*P*		*Weighted %*			*P*		
Region				**0.043**					**0.014**		
Addis Ababa	78	67.3	32.7		1.51 (0.87–2.64)	67.8	26.9	5.3		1.97 (1.03–3.78) *	0.73 (0.40–1.33)
Afar	44	84.4	15.6		0.58 (0.20–1.69)	84.4	3.7	11.9		0.22 (0.09–0.51)**	1.20 (0.31–4.55)
Amhara	437	64.1	35.9		1.74 (1.22–2.50) **	64.4	20.7	14.9		1.59 (1.01–2.49) *	2.02 (1.27–3.20) **
Oromia^b^	941	75.7	24.3		Ref	76.0	15.4	8.6		Ref	Ref
SNNP	521	73.4	26.6		1.13 (0.70–1.82)	73.6	12.9	13.5		0.8 (0.52–1.41)	1.59 (0.81–3.11)
Tigray	147	72.4	27.6		1.19 (0.79–1.77)	72.8	18.3	9.3		1.24 (0.74–2.08)	1.09 (0.62–1.92)
Residence				**0.049**					**0.003**		
Urban	463	67.6	32.4		Ref	67.7	22.8	9.6		Ref	
Rural	1,706	73.8	26.2		0.74 (0.55–1.00)	74.1	14.4	11.5		0.58 (0.42–0.81) **	0.12 (0.73–1.70)
Wealth				**0.006**					** < 0.001**		
Lowest Quintile	430	81.9	18.1		Ref	82.4	6.5	11.1		Ref	REF
Lower Quintile	426	70.8	29.2		1.86 (1.22–2.85) **	70.8	18.4	10.8		3.28 (1.91–5.64) ***	1.07 (0.59–1.94)
Middle Quintile	458	71.2	28.8		1.83 (1.21–2.85) **	71.2	15.2	13.6		2.69 (1.52–4.75) ***	1.35 (0.83–2.18)
Higher Quintile	428	71.0	29.0		1.85 (1.20–2.84) **	71.7	18.7	9.6		3.32 (1.80–6.15) ***	1.03 (0.60–1.74)
Highest Quintile	426	67.5	32.5		2.17 (1.46–3.25) ***	67.5	22.6	10.0		4.22 (2.47–7.21) ***	1.04 (0.62–1.74)
Relationship Status				0.333					**0.009**		
Married	2,106	72.6	27.4		Ref	72.9	16.4	10.7		Ref	
Cohabitating	63	66.3	33.7		1.35 (0.73–2.50)	66.3	10.3	23.5		0.69 (0.29–1.64)	2.35 (1.19–4.64)
Relationship history				**0.019**					**0.001**		
In first union	1,891	73.4	26.6		Ref	73.7	16.4	10.0		Ref	Ref
Had prior union	278	66.2	33.8		1.41 (1.06–1.87) *	66.2	15.1	18.7			
Polygyny				0.198					0.153	1.02 (0.68–1.55)	2.02 (1.38–2.95) ***
No	1,967	72.3	28.0		Ref	72.3	16.8	10.9		Ref	Ref
Yes	202	76.8	23.2		0.78 (0.53–1.14)	76.8	10.9	12.3		0.61 (0.37–1.01)	1.03 (0.61–1.74)
Age				**0.008**					**0.011**		
15–19	231	65.5	34.5		Ref	66.6	17.7	15.7		Ref	Ref
20–24	509	67.9	32.1		0.90 (0.60–1.35)	68.0	19.5	12.6		1.06 (0.67–1.66)	0.73 (0.41–1.30)
25–29	634	72.5	27.5		0.72 (0.48–1.08)	72.7	18.1	9.2		0.92 (0.56–1.52)	0.51 (0.28–0.91) *
30–34	418	77.8	22.2		0.49 (0.30–0.79) *	77.8	10.8	11.4		0.51 (0.29–0.92)	0.57 (0.29–1.14)
35–39	286	79.6	20.4		0.49 (0.30–0.79) **	80.0	12.4	7.6		0.57 (0.32–1.02)	0.39 (0.19–0.81) *
40–49	91	68.3	31.7		0.88 (0.44–1.75)	68.3	18.4	13.3		1.00 (0.42–2.35)	0.76 (0.32–1.83)
Parity				** < 0.001**					**0.002**		
Nulliparous	508	64.9	35.1		Ref	65.5	22.5	12.0		Ref	Ref
1 child	421	68.5	31.5		0.85 (0.60–1.19)	68.5	19.0	12.4		0.80 (0.55–1.16)	0.93 (0.57–1.51)
2–3 children	569	75.0	25.0		0.62 (0.44–0.86) **	75.2	15.1	9.7		0.58 (0.41–0.82) **	0.68 (0.41–1.14)
4 or more children	578	78.5	21.5		0.51 (0.36–0.72) ***	79.5	10.6	10.7		0.40 (0.26–0.58) ***	0.72 (0.43–1.19)
Religion				0.162					0.069		
Orthodox	804	69.0	31.0		Ref	69.2	20.7	10.1		Ref	
Muslim	735	76.4	23.6		0.69 (0.48–0.98) *	76.8	13.0	10.2		0.57 (0.37–0.88) *	0.91 (0.58–1.42)
Protestant/Other^a^	588	72.4	27.6		0.85 (0.57–1.26)	72.6	14.1	13.4		0.67 (0.44–1.02)	1.21 (0.67–2.17)
Education				0.119					**0.004**		
Never Attended	912	74.7	25.3		Ref	75.0	13.5	11.5		Ref	
Primary	862	70.1	29.9		1.26 (0.95–1.66)	70.4	16.7	12.9		1.32 (0.93–1.85)	1.19 (0.80–1.77)
Secondary	238	69.0	31.0		1.32 (0.90–1.94)	69.0	22.7	8.3		1.81 (1.22–2.68) **	0.77 (0.42–1.41)
More than Secondary	157	77.6	22.4		0.85 (0.55–1.31)	77.6	19.3	3.1		1.37 (0.87–2.18)	0.26 (0.11–0.59)
Gestational Age, mean	2,169	5.37	5.18	**0.029**	0.94 (0.90–99) *	5.37	5.19	5.15	**0.036**	0.95 (0.89–1.01)	0.93 (0.87–1.00)

*P*-value based on design-based *F* statistics. Bold indicates *p* < 0.05

^a^Other religion (*n* = 27) includes Catholic, Traditional, Wakefeta, No religion/non-believer, and “Other”

^b^Oromia was selected as the reference region because it is the largest region by sample size and has moderate levels of both any and severe RC, providing a neutral and statistically stable baseline for comparison across regions

^***^ *p* < 0.001, ** *p* < 0.01, ** *p* < 0.05

When RC was analyzed by severity, the experiences of less severe and more severe RC varied across sociodemographic groups (Table 3). For example, the prevalence of less severe RC ranged from 3.7% in Afar to 26.9% in Addis Ababa, while more severe RC ranged from 5.3% in Addis Ababa to 14.9% in Amhara (*p* = 0.014). More severe RC was reported more frequently among rural women than urban women (11.5% vs. 9.6%; *p* < 0.01). Experience of less severe RC generally increased with household wealth, ranging from 6.5% among the poorest households to 22.6% among the wealthiest households (*p* < 0.003). About a quarter (23.5%) of women cohabitating with their partner experienced more severe forms of RC compared to one in ten (10.7%) married women (*p* = 0.009). A significantly greater proportion of women who had a previous serious relationship before their current one experienced more severe RC compared to in their first union (18.7% vs. 10.0%, *p* = 0.001). Less severe RC varied by age, with approximately one in five women aged 15–29 or 40–49 reporting verbal discouragement of family planning compared to one in ten women aged 30–39 (*p* = 0.011). The youngest (ages 15–19) and the oldest women (ages 40–49) experienced the highest prevalence of more severe RC (15.7% and 13.3%, respectively). Experience of less severe RC decreased with increasing parity, from 22.5% among nulliparous women to 10.6% among women with four or more children (*p* = 0.002). Nulliparous and primiparous women experienced the highest proportion of more severe RC (12.0% and 12.4%, respectively). Among religious groups, 20.7% of Orthodox women experienced verbal discouragement of family planning, compared to 13.0% of Muslim women and 14.1% of women with Protestant/Other religious affiliation; these differences were marginally significant (*p* = 0.069). Women with higher education reported less severe RC (primary: 16.7%; secondary: 22.7%) more frequently than those who never attended school (13.5%; p = 0.004). Conversely, the most severe forms of RC decreased with higher educational attainment, from 11.5% among women who never attended school to 3.1% among women with more than a secondary education (*p* = 0.004). There were no statistically significant differences in RC severity categories by polygyny status.

### Correlates of reproductive coercion

Sociodemographic correlates associated with increased odds of experiencing any RC in the past year included higher household wealth [adjusted odds ratio (aOR) _lower quintile_ = 1.93, 95% CI: 1.25–2.97, *p* < 0.001; aOR _highest quintile_ = 2.57, 95% CI: 1.50–4.13, *p* < 0.001] relative to the poorest households, and residence in Amhara compared to Oromia (aOR = 1.82, 95% CI: 1.21–2.75, *p* < 0.01) (Table 4). Having a previous marriage or cohabitating relationship was not significantly associated with increased odds of any RC (aOR = 1.24, 95% CI: 0.94–1.64, *p* = 0.133). Women with two or more children had lower odds of experiencing any RC compared to nulliparous women (aOR _2–3 children_ = 0.57, 95% CI: 0.41–0.80, *p* < 0.05; aOR _4 or more children_ = 0.49, 95% CI: 0.34–0.72, *p* < 0.001). Having more than a secondary education was also associated with a 61% reduction in odds of any RC (aOR = 0.39, 95% CI: 0.22–0.64, *p* < 0.001). Relationship status, polygyny, and religion were not significantly associated with any RC before pregnancy.

**Table 4:** Multivariable associations of sociodemographic characteristics with reproductive coercion in the 12 months before pregnancy (*N* = 2169)

	Model 1 **ANY RC** (*binary RC*)	Model 2 **RC Severity** (*categorical RC*)	
	***Yes**** (N* = *602)* *Vs* *No (N* = *1567)*	***Less Severe**** (N* = *365)* *Vs* *None (N* = *1567)*	***More Severe**** (N* = *237)* *Vs* *None (N* = *1567)*
	aOR (95% CI) ^a^	aRRR (95% CI)^a^	
Region (Ref: Oromia)^c^			
Addis Ababa	1.17 (0.63–2.20)	1.23 (0.60–2.52)	0.66 (0.32–1.40)
Afar	0.82 (0.24–2.75)	**0.35 (0.13–0.94)** *	1.55 (0.38–6.29)
Amhara	**1.82 (1.21–2.75)** **	1.56 (0.92–2.64) †	**2.40 (1.34–4.04)** **
SNNP	1.09 (0.69–1.71)	0.91 (0.55–1.51)	1.33 (0.73–2.42)
Tigray	1.29 (0.81–2.05)	1.17 (0.67–2.04)	1.55 (0.79–3.03)
Household wealth (Ref: Lowest quintile)			
Lower quintile	**1.93 (1.25–2.97)** **	3.46 (2.05–5.84)***	1.15 (0.61–2.17)
Middle quintile	**1.85 (1.24–2.77)** **	2.68 (1.57–4.67)***	1.46 (0.87–2.44)
Higher quintile	**1.93 (1.26–2.95)** **	3.35 (1.89–5.93)***	1.09 (0.60–1.99)
Highest quintile	2.57 (1.50–4.13)***	4.16 (2.32–7.44)***	1.85 (0.95–3.59) †
Relationship status (Ref: Married)			
Living with partner as if married	1.01 (0.52–1.98)	0.47 (0.19–1.13) †	**2.04 (1.06–3.94)** *
Relationship history (Ref: In first union)			
Had prior marriage or cohabitating relationship	1.24 (0.94–1.64)	0.89 (0.58–1.38)	**1.84 (1.22–2.78)** **
Polygyny (Ref: Monogamous Union)			
Yes	1.02 (0.69–1.50)	0.98 (0.57–1.68)	1.09 (0.66–1.79)
Parity (Ref: Nulliparous)			
1 child	0.80 (0.57–1.14)	0.78 (0.53–1.16)	0.88 (0.52–1.48)
2–3 children	**0.57 (0.41–0.80)** *	**0.60 (0.42–0.87)** **	**0.54 (0.32–0.91)** *
4 or more children	0.49 (0.34–0.72)***	0.42 (0.27–0.68)***	0.59 (0.34–1.03) †
Religion (Ref: Protestant/Other^b^)			
Orthodox	0.83 (0.53–1.32)	1.11 (0.65–1.88)	**0.51 (0.28–0.94)** *
Muslim	0.72 (0.47–1.10)	0.74 (0.44–1.27)	0.66 (0.39–1.12)
Education (Ref: Never attended)			
Primary	0.95 (0.71–1.28)	0.88 (0.60–1.29)	1.07 (0.67–1.69)
Secondary	0.71 (0.45–1.12)	0.80 (0.48–1.34)	0.55 (0.27–1.12) †
More than secondary	**0.39 (0.22–0.64**) ***	**0.49 (0.27–0.88)** *	0.16 (0.07–0.40)***
Gestational age, mean (SD)	0.95 (0.90–1.00) †	0.96 (0.90–1.02)	0.94 (0.88–1.02)

*OR* odds ratio, *aOR* Adjusted odds ratio, *aRRR* adjusted relative risk ratio, *CI* confidence interval, *Ref* reference category, *SNNP* Southern Nations, Nationalities, and Peoples. Bold indicates* p* < 0.05

^a^Adjusted for variables *p* < 0.20 in bivariate analyses: region, wealth, relationship status, relationship history, polygyny, parity, religion, education, and gestational age

^b^Other religions (*n* = 27) include Catholic, Traditional, Wakefeta, No religion/non-believer, and “Other"

^c^Oromia was selected as the reference region because it is the largest region by sample size and has moderate levels of both any and severe RC, providing neutral and statistically stable baseline for comparison across regions

^***^p < 0.001, ** *p* < 0.01, * *p* < 0.05, † *p* < 0.10

In multinomial models, residing in Afar was protective against less severe RC [adjusted relative risk ratio (aRRR) = 0.35, 95% CI: 0.13–0.94, p < 0.05], while living in Amhara was associated with a 2.5-fold increased risk of more severe RC (aRRR = 2.40, 95% CI: 1.34–4.04, *p* < 0.01) compared to no RC, relative to women living in Oromia. Higher household wealth was associated with a two to four-fold elevated risk of less severe RC relative to the poorest households (aRRR _lower quintile_ = 3.46, 95% CI: 2.05–5.84, *p* < 0.001; aRRR _middle quintile_ = 2.68, 95% CI: 1.57–4.67, *p* < 0.001; aRRR _higher quintile_ = 3.35, 95% CI 1.80–5.93, *p* < 0.001; aRRR _highest quintile_ = 4.16, 95% CI: 2.32–7.44, *p* < 0.001]. Residing in the wealthiest households was marginally associated with a heightened risk of more severe RC compared to no RC relative to women living in the poorest households (aRRR _highest quintile_ = 1.85, 95% CI: 0.95–3.59, *p* < 0.1). Cohabitating relationships were marginally protective against less severe RC (aRRR = 0.47, 95% CI: 0.19–1.13, *p* < 0.1), but associated with double the risk of more severe RC relative to marital unions (aRRR = 2.04, 95% CI: 1.06–3.94, *p* < 0.05). Having a history of previous marriage or cohabitating relationships also showed opposing patterns by RC severity: previous relationships were not associated with less severe RC (aRRR = 0.89, 95% CI: 0.58–1.38, *p* = 0.607), but nearly doubled the risk of more severe RC (aRRR = 1.84, 95% CI: 1.22–2.78, *p* < 0.01) compared to women whose current relationship was their first. Having two or more children was protective against less severe RC compared to no RC, relative to being nulliparous (aRRR _2–3 children_ = 0.60, 95% CI: 0.42–0.87, *p* < 0.01; aRRR _4 or more children_ = 0.42, 95% CI: 0.27–0.68, *p* < 0.001). Similarly, having two or three children was associated with a 46% decreased risk of more severe RC compared to no RC relative to nulliparous women (aRRR _2–3 children_ = 0.54, 95% CI = 0.32–0.91, *p* < 0.01). Likewise, having four or more children was marginally protective against more severe RC (aRRR = 0.59, 95% CI: 0.34–1.03, *p* < 0.1). Finally, having more than secondary education was associated with an 84% reduction in the risk of more severe RC (aRRR = 0.16, 95% CI: 0.07–0.40, *p* < 0.001).

## Discussion

This study adds to the growing evidence on RC in low- and middle-income countries [14, 15, 19, 28, 47–50] by documenting the prevalence and correlates of pre-pregnancy RC in a large, regionally representative cohort of Ethiopian women. Nearly 1 in 3 pregnant women experienced RC in the past year, with about 1 in 5 women experiencing less severe forms (verbal discouragement of family planning), and 1 in 7 experiencing severe forms (e.g., explicit inference, threats, or sabotage). This elevated prevalence, higher than estimates reported in general population samples across sub-Saharan Africa (3%−20%) [14, 19], underscores the preconception period a critical window during which partners may actively exert pressure to achieve pregnancy, shaping women’s reproductive trajectories.

Regional differences were particularly notable. Women in Amhara had higher odds of experiencing any RC, marginally higher relative risk of less severe RC, and more than twice the relative risk of severe RC compared with women in Oromia. These disparities align with broader indicators of reduced autonomy and entrenched patriarchal norms: fewer women in Amhara report deciding their first marriage (15.1% vs. 34.6%), more report husband refusal as a reason for stopping school (36% vs. 18.9%), and men are more likely to justify wife beating (46% vs. 26%) [31]. Total fertility is lower in Amhara (3.7 vs. 5.4), [31] suggesting that, despite lower fertility, women may face greater pressure to comply with their partner's expectations. By contrast, women in Afar had a lower risk of less severe RC relative to Oromia, although no protective association was observed for severe or any RC. Notably, estimates for Afar should be interpreted with caution given the small number of participants from this region and the resulting wide confidence interval. Nonetheless, lower contraceptive use (11.6% vs. 28.6%), greater sexual autonomy (44.9% vs. 34.1% can refuse sex), and more equitable male attitudes toward wife beating [31] may help explain emerging patterns—highlighting how sociocultural norms shape exposure to subtler forms of RC.

Although prior research in sub-Saharan Africa finds wealth generally protective [14], household wealth was positively associated with less severe RC, with women in all but the poorest households facing higher risk, while associations with severe RC were weaker. This pattern mirrors IPV literature showing varying relationships between wealth and violence, sometimes with greater risk among wealthier households [51, 52]. Greater household assets in this context—such as land or livestock—may increase opportunities for subtler forms of partner interference without overt coercion, sabotage, or threats. One possible explanation is that in households with greater agricultural assets, partners may pressure women to bear children for labor and economic support. Additionally, wealthier women may face a higher risk of less severe RC because they are more likely to use contraception [53], creating opportunities for partners to discourage ongoing contraceptive use. In contrast, severe RC may be less common in wealthier households, as partners may rely on indirect strategies rather than overt or aggressive tactics. These findings highlight that household wealth does not always translate to women’s individual reproductive autonomy [54] and suggest that economic motivations for RC warrant further study. These findings should not be interpreted to discourage programs aimed at increasing women’s access to assets and economic empowerment.

Relationship context was particularly important for severe RC. Women living with a partner “as if married” or with prior marriages/cohabitating relationships had elevated risk of severe RC compared with formally married women and those in a first union, while showing marginal protection against less severe RC. Severe RC may thus be concentrated in relational contexts with distinct fertility expectations or power dynamics. Qualitative evidence from sub-Saharan Africa suggests that norms valuing children as marital obligations can drive partners to use RC to maintain status or control, particularly among unmarried or previously partnered women [16]. Women with prior relationships may have children from previous unions [28], which could prompt partners in subsequent relationships to exert more severe reproductive control to ensure biological offspring and lineage continuity. Larger age gaps among remarried women may further exacerbate gender inequities [55]. These findings align with evidence that cohabitating and previously married women face higher risk of IPV [54], underscoring the intersection of relationship history, power dynamics, and reproductive control.

Parity and education were protective, with associations varying by severity. Women with two or more children were consistently less likely to experience both less severe and severe RC, consistent with evidence from Ethiopia, the Democratic Republic of Congo, and Kenya showing higher parity is protective against past-year RC [14, 19], likely reflecting pronatalist norms that increase partner interference among nulliparous women [15–17]. Women with more than secondary education had lower odds of any RC and substantially reduced risk of severe RC, reflecting links between education, delayed marriage, and increased reproductive autonomy [56, 57]. Disaggregating RC by severity clarifies that higher education may particularly reduce exposure to overt forms of partner interference while also limiting subtler forms such as verbal discouragement.

These findings highlight the need for comprehensive strategies to prevent RC before pregnancy and support women who have experienced it. Interventions should address structural and relational determinants by increasing women’s access to secondary and higher education, promoting gender-equitable communication within couples, and engaging men and communities to challenge harmful norms. Healthcare providers should screen for RC during antenatal care, privately assess women’s pregnancy intentions, and offer discreet access to contraception or abortion when desired. Expanding access to medical abortion within prenatal clinics and adapting rights-based models like ARCHES [58, 59] for antenatal and postnatal care can provide frameworks for counseling, referral to IPV services, and discreet reproductive health support, prioritizing the reproductive autonomy and safety of women, particularly nulliparous women.

This study has several limitations. First, PMA Ethiopia enrolled women who were pregnant at any gestational age, including some who subsequently experienced miscarriage or termination. However, women with very early pregnancy loss or termination before enrollment were not represented, which may result in a modest underestimate of pre-pregnancy RC prevalence. Second, despite efforts to minimize social desirability bias by training female interviewers to conduct private interviews using best practices for research on violence against women, some underreporting of RC may have occurred, potentially resulting in conservative prevalence estimates and attenuated associations with correlates. Third, while recall bias is possible given the 12-month reporting period, it is likely minimal because RC events tend to be emotionally salient, the recall window is relatively short, and structured, behaviorally specific questions were used to aid in reporting, recall bias may have affected reporting of RC, potentially resulting in underreporting and misclassification that could attenuate associations with correlates. Fourth, while the cross-sectional design limits the ability to establish temporality between RC and correlates, most assessed correlates are demographic characteristics unlikely to have changed over the 12-month assessment period. Finally, partner covariate data are limited in this secondary analysis. Future research should examine partner factors, including age, education, gender-equitable attitudes, and fertility desires, as well as dyadic factors such as relationship satisfaction, communication, balance of power between partners, and expectations to better understand risk factors for RC perpetration.

## Conclusion

In conclusion, pre-pregnancy RC is common among Ethiopian women, with distinct patterns emerging by RC severity, region, relationship context, household wealth, parity, and education. Severe RC is concentrated in specific relational and cultural contexts, while less severe RC may reflect subtler partner interference linked to contraceptive use and household resources. These findings highlight the need for targeted interventions that combine structural, relational, and healthcare strategies to protect women’s reproductive autonomy.

## Data Availability

The dataset supporting the conclusions of this article is publicly available in the Johns Hopkins Research Data Repository [archive.data.jhu.edu/pmaet]. https://www.pmadata.org/data/request-access-datasets

## Abbreviations

EA: Enumeration area
IPV: Intimate partner violence
PMA: Performance Monitoring for Action
RC: Reproductive coercion
SNNP: Southern Nations, Nationalities and Peoples
SRH: Sexual and reproductive health

## Footnotes

1 Administrative boundaries changed shortly after study enrollment. The Sidama Region and South West Ethiopia Region split off from SNNP in 2020 and 2021, respectively. As of 2022, Ethiopia consisted of 11 regional states and two chartered cities. For simplicity, we use the regional states and city administrations as they were when the 2019–2021 PMA Ethiopia cohort was conducted. Additionally, enrollment in Tigray took place prior to the onset of civil conflict in 2020.
